# Pneumoscrotum secondary to pneumothorax: Clinical images

**DOI:** 10.1002/ccr3.5685

**Published:** 2022-04-05

**Authors:** Alain Mwamba Mukendi, Tshisola Miji Kasapato

**Affiliations:** ^1^ Division of Urology Department of Surgery Thelle Moroegane Regional Hospital University of the Witwatersrand Johannesburg South Africa

**Keywords:** blunt trauma, crepitus, intercostal drain, pneumoscrotum, pneumothorax

## Abstract

This clinical image presents an unusual association of pneumoscrotum and pneumothorax in a trauma setting. Clinicians managing chest trauma patients need to be aware of such association. The etiology of pneumoscrotum must be clarified through history, physical examination, and investigations as necessary considering that its management targets the primary cause.

## Graphical abstract

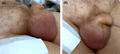

## 1 CASE PRESENTATION

**FIGURE 1:**
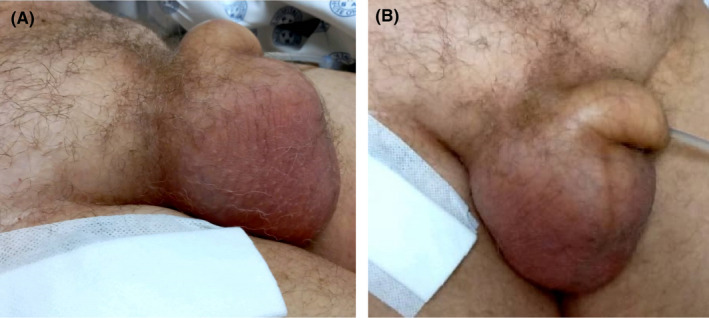
Inguinoscrotal swelling ((A) lateral or side view; (B) anterior view)

**FIGURE 2:**
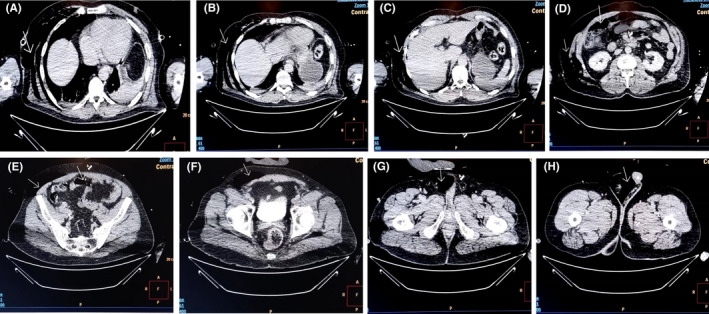
(A–H) Axial CT images demonstrating surgical emphysema (white arrows) overlying the anterolateral and posterior soft tissues of the right chest extending down to the scrotum

**FIGURE 3:**
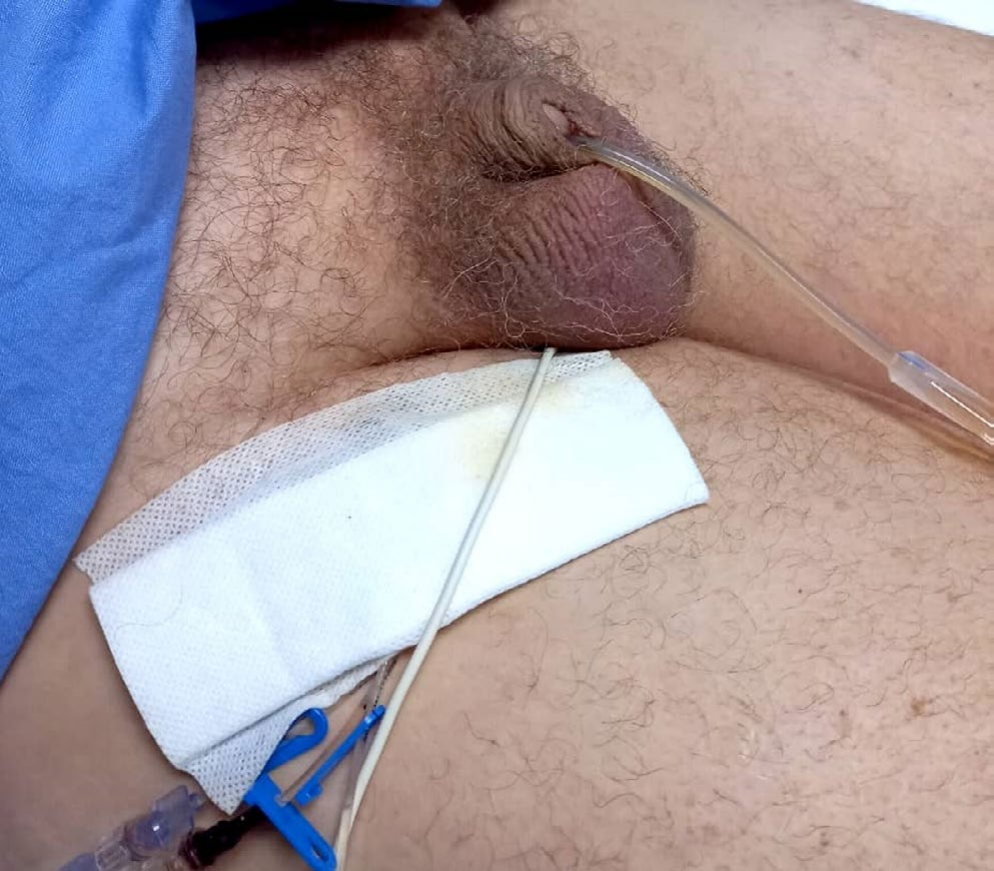
Complete resolution of scrotal swelling after ICD was replaced

A 52‐year‐old male patient was admitted in intensive care unit with polytrauma after a motor vehicle accident. He was intubated on scene for decreased level of consciousness and had an intercostal drain (ICD) inserted for pneumothorax. While awaiting to review CT scan images and report, urology was consulted for rapidly enlarging scrotal swelling querying an infective process. Physical examination revealed a swollen scrotum with crepitus on palpation extending through the inguinal areas to the abdomen (Figure 1A,B) and poor tidaling in the chest tube. Septic markers were all within normal ranges. CT findings included acute intraparenchymal hemorrhage, surgical emphysema overlying the anterolateral and posterior soft tissues of the right chest extending down to the scrotum (Figure 2A,B,C,D,E,F,G,H) and rupture of the external oblique muscle. The ICD was replaced, and the scrotal swelling subsided the following day (Figure 3).

## 2 DISCUSSION

The presence of air within the scrotum (pneumoscrotum) includes scrotal emphysema (surgical/subcutaneous emphysema), which is clinically palpated as crepitus and scrotal pneumatocele (air within the tunica vaginalis) not clinically palpable.^1^, ^2^

The etiology of pneumoscrotum can be intra or extrascrotal. Intra scrotal from gas‐producing bacterial infection or local introduction of air; extrascrotal from pneumoperitoneum, or air accumulation from lungs, mediastinum, or retroperitoneum.^1^, ^2^ In this case, the etiology was extrascrotal (pneumothorax). Identifying the correct etiology is very crucial in the management of this condition.

## CONFLICT OF INTEREST

None.

## AUTHOR CONTRIBUTION

AMM conceived and designed the study, acquired the data, analyzed and interpreted the data, wrote the manuscript, obtained ethics approval, and approved the final manuscript with critical content. TMK acquired, analyzed and interpreted the data, and approved final manuscript.

## ETHICAL APPROVAL

The study was approved by the University of the Witwatersrand Human Research Ethics Committee (HREC Medical); R14/49, Certificate number: M2111161.

## CONSENT

Written informed consent was obtained from the Head of department Dr Kasapato for publication of this manuscript and accompanying pictures as the patient was unconscious until demised and we could not trace any family member. A copy of the written consent is available for review by the Editor‐in‐Chief of this journal.

## Data Availability

The data that support the findings of this study are available from the corresponding author upon reasonable request.
